# Predicting functional outcomes of posterior circulation acute ischemic stroke in first 36 h of stroke onset

**DOI:** 10.1007/s00415-018-8746-6

**Published:** 2018-02-17

**Authors:** Sheng-Feng Lin, Chin-I Chen, Han-Hwa Hu, Chyi-Huey Bai

**Affiliations:** 1 School of Public Health, College of Public Health, Taipei Medical University, Taipei, Taiwan; 2 Department of Neurology, Wan Fang Hospital, Taipei Medical University, Taipei, Taiwan; 3 Department of Neurology, School of Medicine, College of Medicine, Taipei Medical University, Taipei, Taiwan; 4 Graduate Institute of Clinical Medicine, College of Medicine, Taipei Medical University, Taipei, Taiwan; 5 Research Center of Cerebrovascular Disease Treatment, College of Medicine, Taipei Medical University, Taipei, Taiwan; 6 Department of Neurology, Taipei Medical University-Shaung Ho Hospital, Taipei, Taiwan; 7 Department of Public Health, School of Medicine, College of Medicine, Taipei Medical University, 250 Wu-Hsing Street, Taipei City, 110 Taiwan

**Keywords:** Cerebral infarction, Diffusion-weighted imaging, Posterior circulation, National Institutes of Health Stroke Scale (NIHSS), Posterior circulation Alberta stroke program early computed tomography score (PC-ASPECTS)

## Abstract

Posterior circulation acute ischemic stroke constitutes one-fourth of all ischemic strokes and can be efficiently quantified using the posterior circulation Alberta stroke program early computed tomography score (PC-ASPECTS) through diffusion-weighted imaging. We investigated whether the PC-ASPECTS and National Institutes of Health Stroke Scale (NIHSS) facilitate functional outcome prediction among Chinese patients with posterior circulation acute ischemic stroke. Participants were selected from our prospective stroke registry from January 1, 2015, to December 31, 2016. The baseline NIHSS score was assessed on the first day of admission, and brain magnetic resonance imaging was performed within 36 h after stroke onset. Simple and multiple logistic regressions were conducted to determine stroke risk factors and the PC-ASPECTS. Receiver operating characteristics (ROC) curve analysis was performed to compare the NIHSS and PC-ASPECTS. Of 549 patients from our prospective stroke admission registry database, 125 (22.8%) had a diagnosis of posterior circulation acute ischemic stroke. The optimal cutoff for the PC-ASPECTS in predicting outcomes was 7. The odds ratios of the PC-ASPECTS (≤ 7 vs > 7) in predicting outcomes were 6.33 (*p* = 0.0002) and 8.49 (*p* = 0.0060) in the univariate and multivariate models, respectively, and 7.52 (*p* = 0.0041) in the aging group. On ROC curve analysis, the PC-ASPECTS demonstrated more reliability than the baseline NIHSS for predicting functional outcomes of minor posterior circulation stroke. In conclusion, both the PC-ASPECTS and NIHSS help clinicians predict functional outcomes. PC-ASPECTS > 7 is a helpful discriminator for achieving favorable functional outcome prediction in posterior circulation acute ischemic stroke.

**Electronic supplementary material:**

The online version of this article (10.1007/s00415-018-8746-6) contains supplementary material, which is available to authorized users.

## Introduction

Posterior circulation infarction accounts for 20% or more of all acute ischemic stroke cases [5, 11, 14]. Posterior circulation acute ischemic stroke is characterized by mild symptoms of transient neurological attacks of nausea, vomiting, dizziness, and vertigo and moderate to severe symptoms of headache, altered consciousness, bulbar signs of slurred speech and dysphagia, weakness, sensory dysesthesia, and ataxia [16, 22]. However, patients with posterior circulation stroke may exhibit a delayed time to presentation, compared with patients with anterior circulation stroke [4]. Until now, no reliable method has been established for predicting the functional outcome of posterior circulation ischemic stroke.

Routine examinations for patients with stroke include clinical assessment using the National Institutes of Health Stroke Scale (NIHSS) and brain imaging. In regional community hospitals and large medical center hospitals, noncontrast brain computed tomography (CT) remains the most widely performed brain-imaging technique because it rapidly detects hemorrhage, is readily available, and saves time, which are crucial for tissue plasminogen activator administration [9]. Despite being widely used, noncontrast CT is not the optimal method of comprehensively assessing posterior circulation infarction [9].

Brain magnetic resonance imaging (MRI) has advantages of visualization of the posterior fossa, detection of hyperacute infarct lesions within 3–6 h by using a diffusion-weighted imaging (DWI) sequence [7, 9, 17], and a high rate of agreement among physicians for use in the detection of early ischemic lesions [6]. Over the past decade, the posterior circulation Alberta stroke program early computed tomography score (PC-ASPECTS)—a 10-point scoring tool similar to the anterior circulation ASPECTS [3] and having advantages of simplicity and easy application—has been used to evaluate posterior circulation infarction [18, 19, 23]. The extent of early infarction may help physicians predict a patient’s functional outcome.

Although proven to accurately predict the outcome of stroke [1], the NIHSS is weighted for anterior circulation symptoms [10]. Our main goal was to investigate whether the PC-ASPECTS with DWI sequences within 36 h of stroke onset as well as the baseline NIHSS corresponds to the functional outcomes of our Chinese patients.

## Methods

### Study aims

 The specific aims of the present study were as follows: (1) to investigate the unfavorable outcome predictors, (2) to compare the NIHSS and PC-ASPECTS with respect to functional outcome prediction, and (3) to identify the optimal cutoff point for the PC-ASPECTS for predicting favorable and unfavorable functional outcomes. The study protocol was approved by the Joint Institutional Review Board of Taipei Medical University.

### Participants

**Fig. 1:**
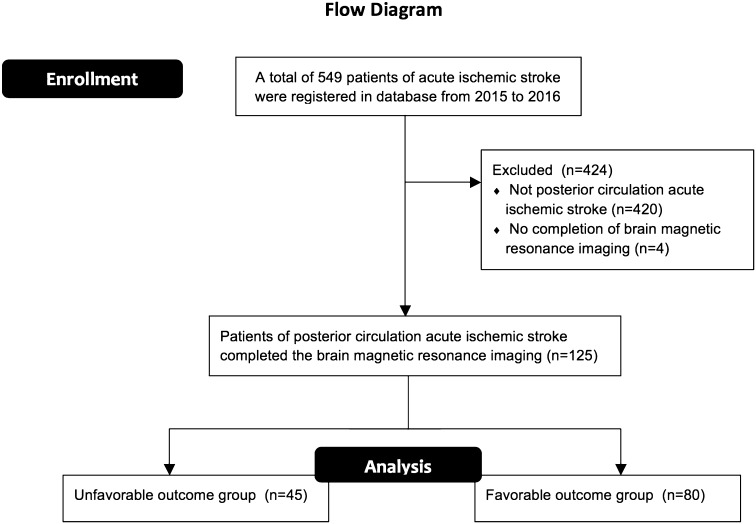
Flow diagram of the enrollment of study participants

Data on the characteristics (age, sex, hypertension, diabetes mellitus, dyslipidemia, and atrial fibrillation) of patients with posterior circulation acute ischemic stroke were retrospectively collected from our prospective stroke admission registry database of the Department of Neurology, Wan Fang Hospital, Taipei Medical University, from January 1, 2015, to December 31, 2016. Three major inclusion criteria were applied in this study: (1) patients were examined by our neurology specialists and had a baseline NIHSS score within the first 24 h of admission; (2) patients presented clinical symptoms and signs characteristic of posterior circulation acute ischemic stroke; and (3) patients underwent brain MRI within 36 h of stroke onset, because an admitted patient is likely to wait up to a maximum of 36 h until brain MRI in our hospital. A study assessed the PC-ASPECTS with imaging performed within 12–36 h [23]. Patients with intracranial hemorrhage, subarachnoid hemorrhage, subdural hemorrhage, epidural hemorrhage, and venous territory infarction and those with both anterior and posterior circulation ischemic stroke were excluded from our analysis. A flow diagram of the enrollment of study participants is shown in Fig. 1.

### Functional outcome assessment

The functional outcomes were assessed at day 90 according to the modified Rankin Scale (mRS) [20], a standardized functional outcome assessment tool with the following score ranges: 0–2, no symptoms to slight disability; 3–5, moderate to severe disability; and 6, death. Patients with mRS scores of 0–2 and 3–5 were categorized into the favorable and unfavorable outcome groups, respectively. An mRS score of 0–2 should be selected as a favorable outcome because the ability to perform complex activities of daily life was the major consideration [25]. All clinical data were reviewed two times to ensure accuracy and completeness.

### Imaging study and PC-ASPECTS scoring

Patients underwent brain MRI on a 1.5-T scanner (Magnetom Avanto; Siemens Medical Solutions, and Horizon LX; GE Healthcare). The DWI, T2- and T1-weighted imaging, and MR arteriography sequences were obtained within the first 36 h of admission. The PC-ASPECTS ranges from 10 to 0, with 10 representing no visible acute ischemic lesion on the DWI sequence of the following anatomical location. One point is deducted for any lesion on either side of the cerebellum, occipital lobe, or thalamus, and two points are deducted for either a midbrain or a pons acute lesion [19]. We applied the PC-ASPECTS for model analysis with the consensus of two stroke neurologists (SFL, HHH). One rater (HHH) was blinded to the clinical information of the patients. The reliability of the PC-ASPECTS was assessed between the two raters using a sample of 30 MRI scans as a separate part of the study (Supplemental Fig. I).

### Statistics

Prior to data analysis, the normality of continuous variables was assessed using the Kolmogorov–Smirnov and Shapiro–Wilk tests. The baseline characteristics of the unfavorable and favorable outcome groups were analyzed using the Student *t* test for continuous variables if variables fulfilled normal distribution, the Mann–Whitney *U* test if variables violated normal distribution, and Pearson’s Chi squared test for categorical variables. Simple and multiple logistic regression analyses were performed to calculate the odds ratio (OR). In the regression analyses, mRS served as the dependent variable of functional outcomes and sex; age (≥ 70 years vs < 70 years); existence of hypertension, diabetes mellitus, atrial fibrillation, and dyslipidemia; and the baseline NIHSS score and PC-ASPECTS served as the independent variables. Receiver operating characteristic (ROC) curve analysis was performed for the NIHSS and PC-ASPECTS models to predict unfavorable outcomes. The Youden index was computed using sensitivity + specificity − 1 to identify the optimal cutoff point [21] for the PC-ASPECTS in predicting unfavorable outcomes. Cohen’s kappa (*κ*) [2] was used to assess the interrater reliability. All analyses were conducted using SAS 9.4 software.

## Results

### Participants

From January 1, 2015, to December 31, 2016, 549 patients with acute ischemic stroke were registered in our database and 129 (22.8%) patients fulfilled the diagnostic criteria for posterior circulation acute ischemic infarction. Four patients were excluded because they did not complete brain MRI. The results of the variable distribution normality assessment are shown in Supplemental Table I. Of these patients, 80 (64%) had a favorable outcome with an mRS score of 0–2 and 45 (36%) had an unfavorable outcome with an mRS score of 3–6 (Table 1). The unfavorable outcome group comprised older patients and exhibited a higher frequency of diabetes mellitus. The average NIHSS and mRS scores were, respectively, 6.3 ± 7.4 and 3.9 ± 0.9 in the unfavorable outcome group and 2.3 ± 0.2 and 1.3 ± 0.6 in the favorable outcome group. Only one patient treated with intravenous thrombolysis for left posterior cerebral artery territory infarction had a favorable outcome.

**Table 1:** Population characteristics (*N* = 125)

Outcome	Unfavorable	Favorable	*p* value
Number (*N*)	45 (36%)	80 (64%)	
Age (years)	77.0 ± 11.6	66.5 ± 13.6	<0.0001*
Age group (years, *N*)			
≤ 59	3	21	
60–69	10	27	
70–79	10	17	
≥ 80	22	15	
Sex			0.1502
Male (*N*)	21	48	
Female (*N*)	24	32	
Hypertension (*N*)	25	50	0.4468
Diabetes mellitus (*N*)	20	20	0.0253
Dyslipidemia (*N*)	22	43	0.6016
Atrial fibrillation (*N*)	9	8	0.1175
Mean baseline NIHSS	6.3 ± 7.4	2.3 ± 0.2	<0.0001*
Median baseline NIHSS (Q1–Q3)^a^	4.0 (2.0–7.0)	2.0 (1.0–4.0)	0.0005
Mean PC-ASPECTS	7.2 ± 2.2	8.5 ± 0.9	<0.0001*
Median PC-ASPECTS (Q1–Q3)^a^	8.0 (6.0–8.0)	8.5 (8.0–9.0)	<0.0001*
Mean mRS	3.9 ± 0.9	1.3 ± 0.6	<0.0001*
Median mRS (Q1–Q3)^a^	4.0 (3.0–4.0)	1.0 (1.0–2.0)	<0.0001*
Admission to intensive care unit	2	1	0.2627
Intravenous thrombolysis	0	1	0.4514

Mean values are expressed as mean ± standard deviation

*mRS* modified Rankin Scale, *N* number, *NIHSS* The National Institutes of Health Stroke Scale, and *PC-ASPECTS* posterior circulation Alberta stroke program early computed tomography score

* Statistical significance (*α* = 0.05)

^a^Mann–Whitney *U* test was used to assess the medians when variables were not normally distributed

### Predictors of favorable outcomes

An age of > 70 years (OR 3.69, *p* = 0.0011) and the presence of diabetes mellitus (OR 2.40, *p* = 0.0270) were significant predictors of unfavorable outcomes (Table 2). A higher baseline NIHSS score (OR 1.31, *p* = 0.0005) and lower PC-ASPECTS (OR 0.53, *p* = 0.0003) were also independent predictors of unfavorable outcomes.

**Table 2:** Predictors of unfavorable outcomes of posterior circulation acute ischemic stroke in 125 patients: univariate and multivariate analyses

Characteristics	Univariate analysis		Multivariate analysis	
	OR (95% CI)	*p* value	OR (95% CI)	*p* value
Age (> vs ≤ 70 years)	3.69 (1.69–8.09)	0.0011^*^	2.84 (1.14–7.09)	0.0250^*^
Sex (male vs female)	0.58 (0.28–1.22)	0.1517		
Hypertension (yes vs no)	1.33 (0.64–2.80)	0.4473		
Diabetes mellitus (yes vs no)	2.40 (1.11–5.21)	0.0270^*^	2.36 (0.94–5.96)	0.0677
Atrial fibrillation (yes vs no)	2.25 (0.80–6.32)	0.1239		
Dyslipidemia (yes vs no)	0.82 (0.40–1.71)	0.6017		
Baseline NIHSS (per score change)	1.31 (1.13–1.53)	0.0005^*^	1.36 (1.13–1.64)	0.0013^*^
PC-ASPECTS (per score change)	0.53 (0.38–0.75)	0.0003^*^		
PC-ASPECTS (≤ 7 vs > 7)	6.33 (2.37–16.90)	0.0002^*^	8.49 (1.85–39.09)	0.0060^*^

*CI* confidence interval, *OR* odds ratio, *PC-ASPECTS* posterior circulation Alberta stroke program early computed tomography score

* Statistical significance (*α* = 0.05)

### ROC curve analysis

**Fig. 2:**
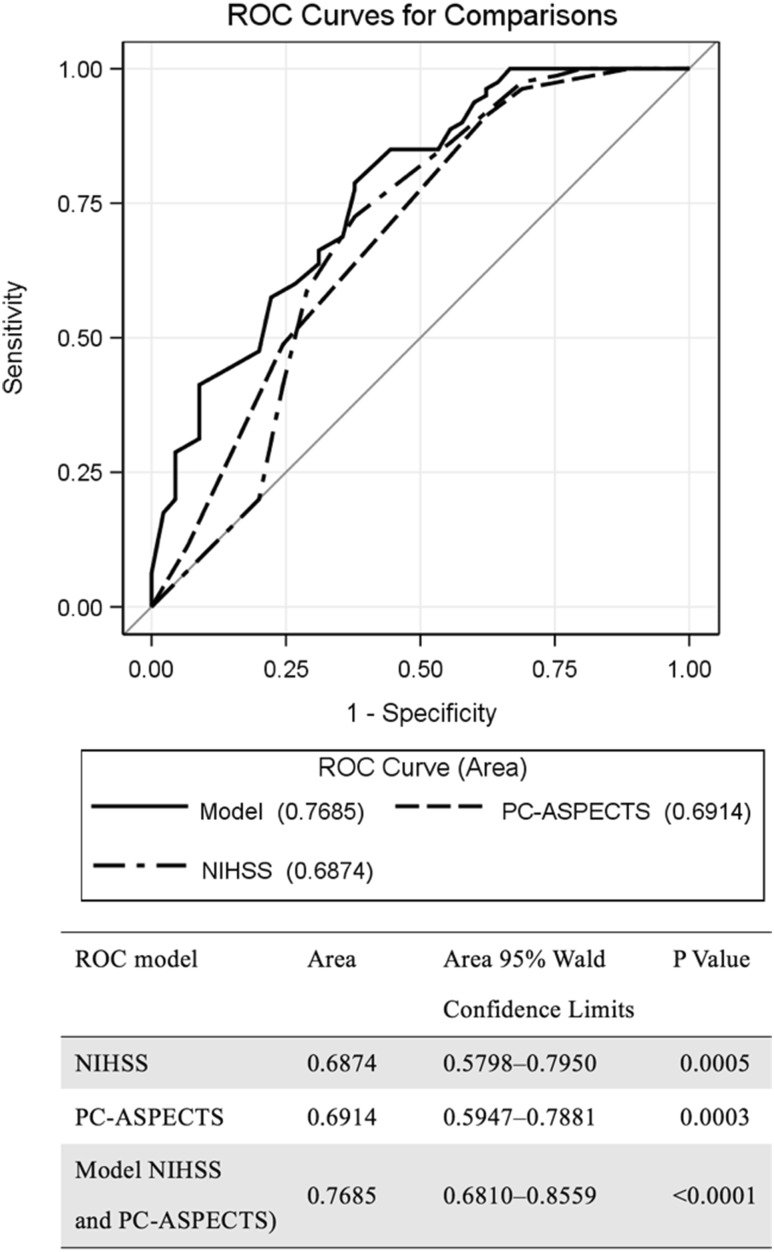
ROC curve analysis of the NIHSS and PC-ASPECTS. *NIHSS* National Institutes of Health Stroke Scale, *PC-ASPECTS* Posterior circulation Alberta stroke program early computed tomography score

In general, the baseline NIHSS score [area under curve (AUC) 0.6874, *p* < 0.0005] and PC-ASPECTS (AUC 0.6914, *p* < 0.0003) were comparable in independent unfavorable outcome prediction. The combination of the baseline NIHSS score and PC-ASPECTS (AUC 0.7685, *p* < 0.0001) had an additive effect in predicting unfavorable outcomes (Fig. 2).

### Optimal cutoff point for the PC-ASPECTS

**Fig. 3:**
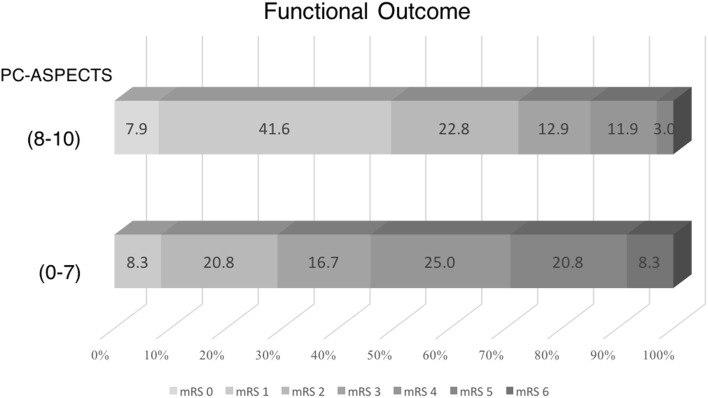
Functional outcomes (percentages) at discharge, according to the modified Rankin Scale (mRS) scores by PC-ASPECTS (≤ 7 vs > 7) grouping

The Youden index of the PC-ASPECTS was 7. Numerous patients (72.3%) in the PC-ASPECTS > 7 group had a favorable outcome and no mortality. Among patients in the PC-ASPECTS ≤ 7 group, 70.8% had an mRS score of 3–6 (Fig. 3). Notably, a PC-ASPECTS of ≤ 7 was the strongest predictor of unfavorable outcomes in both univariate (OR 6.33, *p* = 0.0002) and multivariate (OR 8.49, *p* = 0.0060) analyses (Table 2).

### Age stratification analysis

The age stratification analysis results are presented in Table 3. Among patients aged ≥ 70 years, only the baseline NIHSS score (OR 1.19, *p* = 0.0220) and PC-ASPECTS (OR: 1.84, *p* = 0.0042) were significant predictors of unfavorable outcomes. With the optimal PC-ASPECTS cutoff score of 7, the PC-ASPECTS (≤ 7 vs > 7) was determined to be very effective in predicting unfavorable outcomes (OR 7.52, *p* = 0.0220). By contrast, the PC-ASPECTS did not predict unfavorable outcomes in patients younger than 70 years. Only the baseline NIHSS score and diabetes mellitus were predictors of unfavorable outcomes in these patients.

**Table 3:** Predictors of outcomes of posterior circulation acute ischemic stroke in patients aged < 70 and ≥ 70 years: univariate analysis

Characteristics	< 70-year-old group		≥ 70-year-old group	
	OR (95% CI)	*p* value	OR (95% CI)	*p* value
Sex (male vs female)	0.35 (0.10–1.24)	0.1040	1.13 (0.42–3.04)	0.8018
Hypertension (yes vs no)	0.47 (0.14–1.63)	0.2330	1.00 (0.37–2.71)	1.0000
Diabetes mellitus (yes vs no)	5.38 (1.46–19.84)	0.0114	1.53 (0.54–4.39)	0.4257
Atrial fibrillation (yes vs no)	0.20 (–∞–1.28)^a^	0.6164	1.70 (0.52–5.49)	0.3786
Dyslipidemia (yes vs no)	1.35 (0.39–4.74)	0.6357	0.69 (0.26–1.84)	0.4537
Baseline NIHSS (per score change)	1.84 (1.27–2.67)	0.0013	1.19 (1.03–1.39)	0.0220*
PC-ASPECTS (per score change)	0.69 (0.44–1.06)	0.0882	1.84 (1.27–2.67)	0.0042*
PC-ASPECTS (≤ 7 vs > 7)	3.30 (0.64–17.13)	0.1554	7.52 (1.89–29.84)	0.0041*

*CI* confidence interval, *OR* odds ratio, *PC-ASPECTS* posterior circulation Alberta stroke program early computed tomography score

* Statistical significance (*α* = 0.05)

^a^Result of the exact logistic regression statistic

## Discussion

The PC-ASPECTS and baseline NIHSS help physicians predict an unfavorable outcome, both individually and in combination. In addition to the effect of aging, a PC-ASPECTS of ≤ 7 was the strongest predictor of unfavorable outcomes in our univariate and multivariate models.

A large percentage of patients with a PC-ASPECTS of ≤ 7 had unfavorable outcomes, and this finding is compatible with that of a previous study revealing that a PC-ASPECTS of < 8 was very unlikely to predict favorable outcomes in basilar artery occlusion [19]. The age stratification analysis results reveal that in patients younger than 70 years, the PC-ASPECTS was not a significant predictor of unfavorable outcomes. This result can be attributed to the few young patients with unfavorable outcomes and numerous young patients with acute medullary infarction. The patients with acute medullary infarction were later confirmed as having vertebral artery dissection. In fact, vertebral artery dissection has been recognized as a frequent cause of posterior circulation stroke among young adults [13, 24]. The PC-ASPECTS cannot be accurately assessed in medullary infarction.

On the ROC curve, the AUC determined for the PC-ASPECTS was slightly larger than that for the baseline NIHSS for patients with low NIHSS scores. These patients had a clinical presentation that included symptoms of dizziness, vertigo, neck pain, headache, and signs of Horner syndrome. Although these symptoms and signs are necessary for an acute stroke diagnosis, the NIHSS scoring system does not provide a score for them. Because the NIHSS is weighted more toward anterior circulation symptoms and signs [10], the PC-ASPECTS or MRI is more suitable for the diagnosis and assessment of posterior circulation ischemic stroke [23], particularly in older patients who may not be able to adequately describe their symptoms. Therefore, a combination model of the PC-ASPECTS and baseline NIHSS had an additive effect because the PC-ASPECTS is more powerful in detecting unfavorable outcomes with posterior circulation acute ischemic stroke with an NIHSS score of 0–1 (Supplemental Tables II and III).

For imaging of posterior circulation acute ischemic stroke, MRI with DWI sequences is considered the gold standard for diagnosis [6, 7, 9, 12]. However, in some community hospitals, only CT is available for acute stroke imaging. Nevertheless, non-contrast CT is not recommended for PC-ASPECTS scoring for predicting functional outcomes due to its low sensitivity (0.46, 95% CI 0.37–0.55) to posterior circulation ischemic change [19]. Alternatively, perfusion CT [15] or CT angiography source imaging (CTASI) [18, 19] improved the outcome prediction and facilitated the delineation of the ischemic core when applied to the PC-ASPECTS. For patients with basilar artery occlusion, a PC-ASPECTS of < 8 on perfusion CT [15] and CTASI [18, 19] has been reported to more likely have unfavorable functional outcomes, which is compatible with our result obtained using MRI as an imaging modality. According to our review of the relevant literature, for patients with small artery occlusion in the posterior circulation, no study has investigated whether the PC-ASPECTS is suitable for functional outcome prediction with CT as an imaging modality.

The present study has limitations. Our hospital is located among an aging community; thus, more than half of our patients were older than 70 years. Because these elders had a favorable clinical condition, they were promptly transferred to long-term care facilities under the post-acute care policy in Taiwan [8]. Moreover, because of the retrospective study design involving the review of our own hospital’s stroke registry database, the study was prone to sampling older patients, and data on longitudinal records of functional outcomes after 3 months were unavailable. Nevertheless, our hospital is the only large-scale hospital in the southernmost district of Taipei, Taiwan. Most patients with acute stroke living in southern Taipei usually present to our hospital for admission. With approximately 11.7‰ of the total population in Taiwan, the estimated incidence of acute ischemic stroke is 454 person-years in our district. Because our registry comprised a total of 549 patients from 2015 to 2016, we assumed that more than half of the patients with acute ischemic stroke in this district were admitted to our hospital. We thus consider our enrolled patients as a fairly representative sample of the whole population.

In conclusion, both the PC-ASPECTS and NIHSS help clinicians predict functional outcomes. The PC-ASPECTS is more reliable than the NIHSS in minor stroke prediction. We determined that the combination of the PC-ASPECTS and NIHSS (AUC 0.7685, *p* < 0.0001) has an addictive effect in predicting the functional outcomes of patients with posterior circulation stroke.

## Electronic supplementary material

Below is the link to the electronic supplementary material.

Supplementary material 1 (PDF 150 kb)
