# Supplementary material for: Predicting functional outcomes of posterior circulation acute ischemic stroke in first 36 h of stroke onset
Source: J Neurol. 2018 Feb 17;265(4):926–32. doi: 10.1007/s00415-018-8746-6 (PMC5878189; doi:10.1007/s00415-018-8746-6)
Supplement: Supplementary file 1 — Supplementary material 1 (PDF 150 kb) [file 415_2018_8746_MOESM1_ESM.pdf]

ONLINE SUPPLEMENT

Total number of figures: 1

Total number of tables: 3

Supplemental Figure

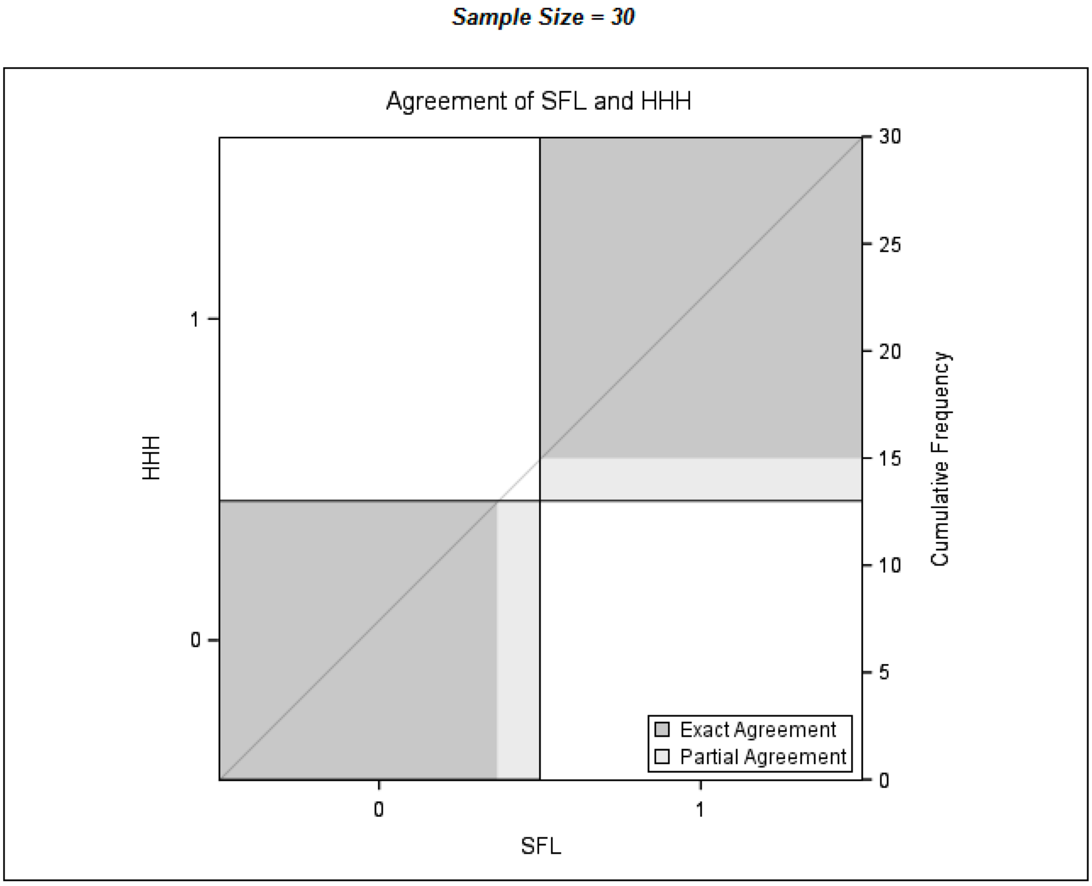

Fig. I Agreement chart of the PC-ASPECTS (≤7 vs >7 score) between two stroke neurologists (SFL, HHH) with a sample of 30 MRI scans. The interrater reliability for the PC-ASPECTS (≤7 vs >7 score) was excellent (κ = 0.87, 95% CI, 0.690–1.000).

Supplemental Tables

Table I. Assessment of continuous variable distribution (N = 125)

Variables	Min.	Max.	Mean ± SD	Kolmogorov–Smirnov		Shapiro–Wilk	
				Statistics	*P* value	Statistics	*P* value
Age (years)	35	95	70.3 ± 13.8	0.0688	>0.1500	0.9797	0.0573
Baseline NIHSS	0	34	3.8 ± 5.0	0.2339	<0.0100*	0.6430	<0.0001*
PC-ASPECTS	0	10	8.0 ± 1.6	0.3002	<0.0001*	0.7723	<0.0001*
mRS	0	6	2.2 ± 1.5	0.2106	<0.0100*	0.8988	<0.0001*

Max., maximum; Min., minimum; mRS, modified Rankin Scale; NIHSS, The National Institutes of Health Stroke Scale; PC-ASPECTS, posterior circulation Alberta stroke program early computed tomography score; SD, standard deviation. *Statistical significance (alpha = 0.05).

Table II. Youden index score for the PC-ASPECTS

PC-ASPECTS	0	1	2	4	5	6	7*	8	9	10
1-Specificity (%)	0	0	0	0	0	3.8	5.0	42.5	37.5	11.3
Sensitivity (%)	97.8	97.8	97.8	97.8	97.8	80.0	93.3	62.2	82.2	93.3

PC-ASPECTS, posterior circulation Alberta stroke program early computed tomography score.

Scores of 0–5 were outlier data.

Table III. Prediction of unfavorable functional outcomes with respect to each baseline NIHSS score

Baseline NIHSS	0	1	2	3	4	5	6	7	8
1-Specificity (%)	20.0	21.3	17.5	13.8	13.8	7.5	3.8	1.3	1.3
Sensitivity (%)	80.0	95.6	95.6	91.1	82.2	91.1	95.6	93.3	95.6
Baseline NIHSS	9	11	12	17	18	19	28	34	
1-Specificity (%)	0.0	0.0	0.0	0.0	0.0	0.0	0.0	0.0	
Sensitivity (%)	97.8	97.8	97.8	95.6	97.8	97.8	97.8	97.8	

NIHSS, National Institutes of Health Stroke Scale
